# Protective Effects of Different Selenium Green Tea Polysaccharides on the Development of Type 2 Diabetes in Mice

**DOI:** 10.3390/foods12234190

**Published:** 2023-11-21

**Authors:** Weilan Gao, Zhan Zheng, Xuehua Wang, Li Wang, Na Zhang, Haiyuan Liu, Xin Cong, Shuyi Li, Zhenzhou Zhu

**Affiliations:** 1 National R&D Center for Se-Rich Agricultural Products Processing, Hubei Engineering Research Center for Deep Processing of Green Se-Rich Agricultural Products, School of Modern Industry for Selenium Science and Engineering, Wuhan Polytechnic University, Wuhan 430048, China; weilangao@126.com (W.G.); 17671055616@163.com (Z.Z.); lwang@whpu.edu.cn (L.W.); 15038327156@163.com (N.Z.); congxinwhpu@whpu.edu.cn (X.C.); shuyi.li198708@gmail.com (S.L.); zhenzhouzhu@126.com (Z.Z.); 2 College of Food Science and Engineering, Wuhan Polytechnic University, Wuhan 430023, China; 3 Enshi Se-Run Material Engineering Technology Co., Ltd., Enshi 445000, China; liuhaiyuan51126@163.com

**Keywords:** tea polysaccharides, selenium-enriched, synthetic selenized, selenium form, type 2 diabetes prevention

## Abstract

Selenium polysaccharides have attracted significant interest due to their superior function to that of individual polysaccharides. However, limited research has compared the protective effects of different selenium polysaccharides from different selenization methods on diabetes. This work aims to compare the preventive effects of natural selenium-enriched green tea polysaccharides (NSe-TPS), synthetic selenized green tea polysaccharides (PCSe-TPS), and a mixture of sodium selenite and green tea polysaccharides (ordinary tea polysaccharides (Ord-TPS)+Se) on the development of diabetes. While establishing a diabetes model induced by a high-sugar, high-fat diet combined with streptozotocin, different selenium polysaccharides were administered daily by gavage for nine weeks. Our findings indicate that PCSe-TPS exhibited superior preventive effects on developing type 2 diabetes compared to NSe-TPS and Ord-TPS+Se. PCSe-TPS effectively regulated glucose metabolism and insulin resistance by activating the PI3K/Akt pathway, thereby preventing elevated blood glucose levels. Additionally, PCSe-TPS mitigated oxidative damage and inflammatory responses in liver tissues. Notably, PCSe-TPS intervention reversed the decline in bacterial species richness and the abundance of *unclassified_Oscillospiraceae* during the development of diabetes in mice. These results provide valuable insights into the protective effects of PCSe-TPS against diabetes development, highlighting its advantages over NSe-TPS and Ord-TPS+Se.

## 1. Introduction

According to the International Diabetes Federation, by 2022, 537 million adults worldwide, or one in ten adults, will be living with diabetes [1]. Diabetes mellitus has emerged as a significant chronic disease, ranking third in terms of threatening human health, following cancer and cardiovascular diseases. Approximately 95% of diabetes is type 2 diabetes (T2D) [2]. Insulin resistance (IR), mainly due to dysregulation of insulin signaling pathways, is generally considered to be the crucial pathogenesis of T2D. Impaired glucose and lipid metabolism, pancreatic islet cell damage, oxidative stress, inflammation, and weight loss are the typical features of T2D [3]. The main modifiable risk factors for T2D are poor diet, sedentary lifestyle, and obesity [4]. Previous studies have found a negative association between T2D and consuming fish, low-fat dairy products, nuts, and fresh fruits and vegetables [5]. This suggests that dietary intervention may slow or even halt the progression of T2D; therefore, using functional foods is a recommended strategy for preventing the onset of diabetes. Furthermore, adopting proactive measures to prevent the development of diabetes is more straightforward and more effective than treating the disease after it has already manifested. 

Selenium (Se), an essential trace element, is known to have implications for T2D risk, with both inadequate and excessive dietary selenium intake found to increase this risk [6]. The World Health Organization recommends 60–200 mg of Se per day for healthy adults, with a maximum tolerable intake of 400 mg [7]. There are two natural forms of Se: inorganic and organic. Organic Se has been reported to have a higher toxicity tolerance dose and bioavailability than inorganic Se [8]. Selenium polysaccharides, consisting of Se and polysaccharides, are an essential form of organic Se. These selenium polysaccharides can be classified into two types: natural selenium polysaccharides derived from selenium-containing plants or microorganisms and synthetic selenium polysaccharides synthesized by the selenization reaction of ordinary polysaccharides and selenium [9]. The selenium content in ordinary selenium-enriched tea polysaccharides is around 0.1–10 mg/kg, while the selenium content in synthetic selenized tea polysaccharides can be up to 10–100 times higher [9]. A recent study found that ordinary and selenium-enriched black tea showed similar alleviation effects on IR and hyperglycemia [10]. Meanwhile, another study found that the selenized polysaccharides were more effective in inhibiting α-amylase and α-glucosidase than individual polysaccharides from *Ribes nigrum* L. [11]. In addition, a recent study showed that the structural characteristics and α-glucosidase inhibition activity of tea polysaccharides (TPS) were significantly affected by different methods of selenization [12]. These findings suggest that the dose of selenium or different selenization methods of polysaccharides are essential for their hypoglycemic effect. However, few studies have compared the hypoglycemic effect of different Se polysaccharides from different artificial selenization methods in vivo, primarily a comprehensive comparison between the hypoglycemic effects of the ordinary selenium-enriched, synthetic selenized and mixed-selenium polysaccharides.

Therefore, this study aimed to compare the protective effects of selenium-enriched, synthetic selenized, and mixed-selenium green tea polysaccharides on the development of T2D in mice. Our study explores the mechanisms of different selenium green tea polysaccharides in preventing the onset of diabetes and their relationships and differences and provides a theoretical basis for utilizing synthetic selenized green tea polysaccharides for special populations. 

## 2. Materials and Methods

### 2.1. Preparation of Different Selenium Green Tea Polysaccharides 

Ordinary green tea and naturally selenium-enriched green tea were purchased from Blue Baked Tea Co., Ltd. (Enshi, Hubei, China). The selenium content of the selenium-enriched tea was 2.16 ± 0.08 μg/g, while that of the ordinary tea was 0.06 ± 0.01 μg/g. Ordinary tea polysaccharides (Ord-TPS) and natural selenium-rich tea polysaccharides (NSe-TPS) were prepared according to our previous study [13]. The chemically synthetic selenized tea polysaccharide with pulsed electric fields (PCSe-TPS) was prepared based on the last study with minor modifications [14]. In brief, Ord-TPS (0.5 g) was dissolved in 50 mL HNO3 (0.5%, *v*/*v*), then Na_2_SeO_3_ (0.5 g) was added. Pulsed electric fields (5 kV/cm/30 °C/4 times) were introduced to promote selenylation. The mixture was cooled (room temperature), and the pH value was adjusted to 6–7 (NaHCO_3_, 1 mol/L). The solution was collected and dialyzed (Mw cutoff: 3500 Da, Beijing Solarbio Technology Co. Ltd., Beijing, China) with ultrapure water for 48 h to remove the unreacted reagents until the solution was colorless when ascorbic acid was added. Afterward, four times the volume of anhydrous ethanol was added to precipitate the tea polysaccharides. The precipitates were then concentrated and lyophilized to obtain synthetic selenized polysaccharides (PCSe-TPS). The selenium content of PCSe-TPS was 246.93 ± 3.95 μg/g. The inorganic selenium-mixed polysaccharide (Ord-TPS+Se) was prepared by mixing Ord-TPS and Na2SeO3 according to the ratio of polysaccharide and selenium in the PCSe-TPS.

### 2.2. Animal Experiment

C57BL/6 male mice (6-week-old) were obtained from the Hubei Province Laboratory Animal Research Center and housed under standard pathogen-free conditions with a 12 h dark/light cycle (23 °C, 55 ± 5% humidity). After one week of adaptation, the mice were divided into seven groups (n = 12): normal control (NC), diabetes model control (DC), positive control (MET), Ord-TPS, NSe-TPS, PCSe-TPS, and Ord-TPS+Se (Figure S1). The NC group was fed a basic diet, while the other groups were fed a high-sucrose and high-fat diet (formula: basic feed + 10% lard + 20% sucrose + 2.5% cholesterol + 1% sodium cholate, the diet provided 3.941 kcal/g, of which 15.6% was from protein, 31.1% from fat, and 53.3% from carbohydrate.). The MET was gavaged with metformin, and the intervention groups were gavaged with different selenium tea polysaccharides (200 mg/kg, the dose chosen through a preliminary experiment (Figure S2)) for six weeks. The daily selenium intake of mice in the SSe-TPS and Ord-TPS+Se groups was calculated via adults (70 kg, 400 μg/kg) by body surface area [15]. After intervention for seven weeks, the mice received intraperitoneal injections of streptozotocin (STZ) for two consecutive days at 100 mg/kg according to the previous study with minor modification [16], and the NC group was administered the exact dosage of sodium citrate buffer. Following two weeks of intraperitoneal injection, fasting blood glucose (FBG) was measured in mice after 12 h of fasting. The FBG values of all mice in the DC group were above 11.1 mmol/L, suggesting that the diabetic mice model was successfully established. At the end of the experiment, the FBG and oral glucose tolerance test (OGTT) were measured; samples of cecum content, plasma, liver, pancreas, and other internal organs were collected. Fresh organs were weighed promptly to calculate the organ index, as described in (1).

(1) Organ index=organ wet weight/body weight of mice

Animal experiments were conducted at the Hubei Province Laboratory Animal Research Center (Hubei, China). This project passed the ethical review of laboratory animal welfare by the Hubei Center for Disease Control and Prevention and the Laboratory Animal Management and Use Committee, and the approval number is Safety Evaluation Center dynamic (Fu) No. 202210207. Experiments were conducted in compliance with national regulations and local guidelines.

### 2.3. Biochemical Analysis

All mice were fasted for 12 h the night before the OGTT test. Mice were given glucose (1.0 g/kg) according to body weight as described in a previous study [17], and the blood glucose values were measured at 30, 60, and 120 min. The changes in blood glucose concentration and the area under the curve (AUC) of the blood glucose at each time point were observed. The formula is shown in Equation (2):

(2) AUC (mmol/L)=(0.5(A+B)+1.5(B+C))/2

where A, B, and C represent the blood glucose values at 0, 30, and 120 min, respectively.

The serum insulin content (INS) was measured using an enzyme-linked immunosorbent assay (ELISA). INS ELISA kit was purchased from Jiangsu Enzyme Immunoassay Industry Co., LTD (Yancheng, China), and the insulin resistance index (HOMA-IR) was evaluated using the provided Equation (3) below.

(3) HOMA−IR=(FBG×INS)/22.5

where FBG represents the fasting blood glucose (mmol/L), and INS stands for insulin content (mIU/L).

Serum triglyceride (TG), total cholesterol (TC), high-density lipoprotein cholesterol (HDL-C), and low-density lipoprotein cholesterol (LDL-C) were measured using a serum biochemistry analyzer. The enzyme activities of aspartate aminotransferase (AST), alanine aminotransferase (ALT), and alkaline phosphatase (ALP) were measured using ELISA. Kits were purchased from Radu Life Science Co., Ltd. (Shenzhen, China).

### 2.4. Histopathological Analysis of the Pancreas and Liver

The pancreas and a portion of the liver were fixed in paraformaldehyde (4%), dehydrated in gradient ethanol, washed in xylene, embedded in paraffin, sectioned (4 μm), and baked. Afterward, the tissue sections were stained with hematoxylin and eosin and observed under a microscope.

### 2.5. Measurement of Oxidative Stress and Inflammatory Parameters in the Liver

The superoxide dismutase (SOD), glutathione peroxidase (GSH-Px), malondialdehyde (MDA), and total antioxidant capacity (T-AOC) in the liver homogenate were detected by commercial kits and following the manufacturers’ instructions. Determination kits were purchased from Nanjing Jiancheng Bioengineering Institute (Nanjing, China). The protein expression levels of interleukin-1β (IL-1β), interleukin-6 (IL-6), and tumor necrosis factor-α (TNF-α) in the liver homogenate were determined using ELISA; ELISA kits were purchased from Jiangsu Enzyme Immunoassay Industry Co., Ltd. (Yancheng, China).

### 2.6. Real-Time Quantitative PCR

Liver tissue RNA was extracted using the kit (Novica Zhan Biotechnology Co., LTD., Nanjing, China). The quality of the RNA was estimated using the OD260/OD280 ratio. The experimental requirements were met if the ratio was between 1.8 and 2.0. The total RNA was stored in a refrigerator at −80 °C until use. Reverse transcribed into cDNA using a two-step process. In the first step, the gDNA was removed. The reaction system contained 4 μL of 4× gDNA wiper rMix and 4 μg of total RNA added to 16 μL of nuclease-free water, which was gently blown and mixed with a pipetting gun. The reaction was performed (42 °C, 2 min). RNA was reverse transcribed in the second step, and the reaction system consisted of 4 μL of 5× HiScrip II qRT SuperMix II and the first step reaction solution. The cDNA was obtained using the following conditions: 50 °C: 15 min→85 °C: 5 s. The reaction system, reaction procedure, and primers are shown in supplementary materials. (Tables S1–S3). The relative quantification was calculated by the comparative 2^–∆∆CT^ method.

### 2.7. Gut Microbiota Analysis

Total DNA was extracted from the cecal contents, and the DNA extraction quality was assessed using electrophoresis on a 1% agarose gel. Subsequently, the v3-v4 region of the 16S rRNA gene was amplified using PCR with the forward primer 338F (5′-ACTCCTACGGGAGGCAGCA-3’) and the reverse primer 806R (5′-GGACTACHVGGGTWTCTAAT-3’). The Illumina NovaSeq6000 platform was employed for constructing a small fragment library for sequencing. The obtained sequences underwent quality filtering using Trimmomatic v0.33 software. Primer sequences were identified and removed with Cutadapt 1.9.1 software, resulting in the acquisition of high-quality sequences. QIIME2 2020.6 was used for sequence denoising, followed by eliminating chimeric sequences. Subsequently, operational taxonomic units (OTUs) were identified at a 97% similarity threshold. Sequencing data were deposited in the NCBI Sequence Read Archive (SRA) (http://www.ncbi.nlm.nih.gov/sra/ (accessed on 16 October 2023)) under the Bioproject ID PRJNA1028394. The α diversity index of the samples, encompassing the Chao1, Ace, Simpson Index, Shannon Index, and Rarefaction Curves, was evaluated using QIIME software. To compare the β diversity of species among different samples, principal coordinate analysis (PCoA) was performed. Lastly, linear discriminant analysis effect size (LEfSe) and linear discriminant analysis (LDA) were conducted to identify biomarkers with statistically significant differences between various groups.

### 2.8. Statistical Analysis

Results are expressed as mean ± standard error of the mean (SEM). The Kruskal–Wallis test is performed to compare medians between nonnormally distributed groups. Comparisons among groups were performed by one-way analysis of variance (ANOVA) followed by the Turkey post hoc test and graphed by GraphPad Prism 8.

## 3. Results

### 3.1. Protective Effects of Different Selenium Tea Polysaccharides on Basic Physiological Indices in the Development of Diabetic Mice

**Figure 1:**
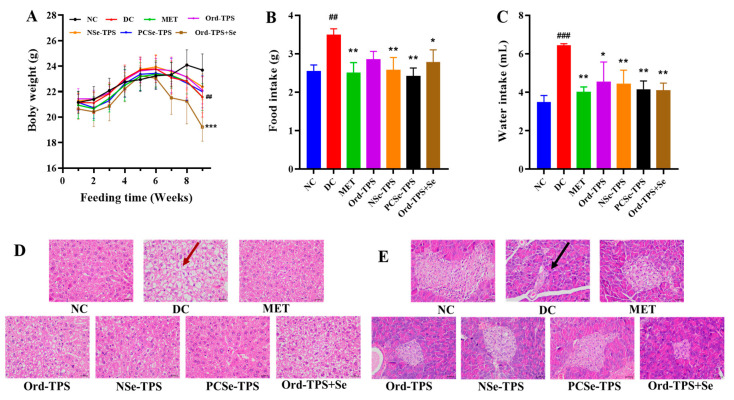
Effects of different selenium green tea polysaccharides on (**A**) body weight (n = 12), (**B**) food intake (n = 12), (**C**) water intake (n = 12), (**D**) liver, and (**E**) pancreas histopathology in mice (n = 3). Cytoplasmic vacuolization (red arrow) and β-cell count (black arrow). The magnification was 400×, and the ruler was 100 µm. NC: normal control; DC: diabetes model control; MET: metformin positive control; Ord-TPS: ordinary tea polysaccharides; NSe-TPS: natural selenium-rich tea polysaccharides; PCSe-TPS: the chemically synthetic selenized tea polysaccharide with pulsed electric fields; Ord-TPS+Se: Ord-TPS and Na_2_SeO_3_ mixture. ## *p* < 0.01, ### *p* < 0.001, compared to normal control (NC) group, * *p* < 0.05, ** *p* < 0.01, *** *p* < 0.001 compared to diabetes model (DC) group.

After STZ injection at week 7, a significant decrease in body weight was observed in mice, followed by significantly higher food and water intake in the DC group compared to the NC group at week 9, indicating typical symptoms of diabetes [4] (Figure 1). The positive control group and different selenium tea polysaccharides groups showed promising inhibition of body weight loss, polydipsia, and polyphagia (Figure 1). The DC group exhibited significantly increased indices of the liver, kidney, and pancreas (Table 1), suggesting swelling and dysfunction of these organs in diabetic mice. The supplementation of different selenium tea polysaccharides demonstrated improvements in the mentioned symptoms (Table 1), corroborating the liver and pancreas histopathological analysis. As shown in Figure 1D, histological examination revealed hepatocyte nuclei swelling, disordered arrangement, cytoplasmic vacuolization (black arrow), and hepatic steatosis in the DC group. However, the selenium tea polysaccharide groups showed less degeneration of nuclei and inflammatory infiltration, with the best protective effect observed in the PCSe-TPS group. Furthermore, the intervention of selenium tea polysaccharides demonstrated a notable mitigation of the reduction in β-cell count and the degeneration and cavitation of islets. These beneficial effects were particularly pronounced in the PCSe-TPS and NSe-TPS groups (Figure 1E).

It was observed that the Ord-TPS+Se group exhibited a more pronounced weight loss compared to the other groups at week 9 (*p* < 0.001) (Figure 1A). Moreover, the heart, spleen, and lung indices were significantly elevated in the Ord-TPS+Se group compared to the NC and DC groups (Table 1). This phenomenon could be attributed to the prolonged consumption of inorganic selenium, which has a higher toxicity level than organic selenium [8].

### 3.2. Protective Effects of Different Selenium Tea Polysaccharides on Glucose and Lipid Metabolism in the Development of Diabetic Mice

**Figure 2:**
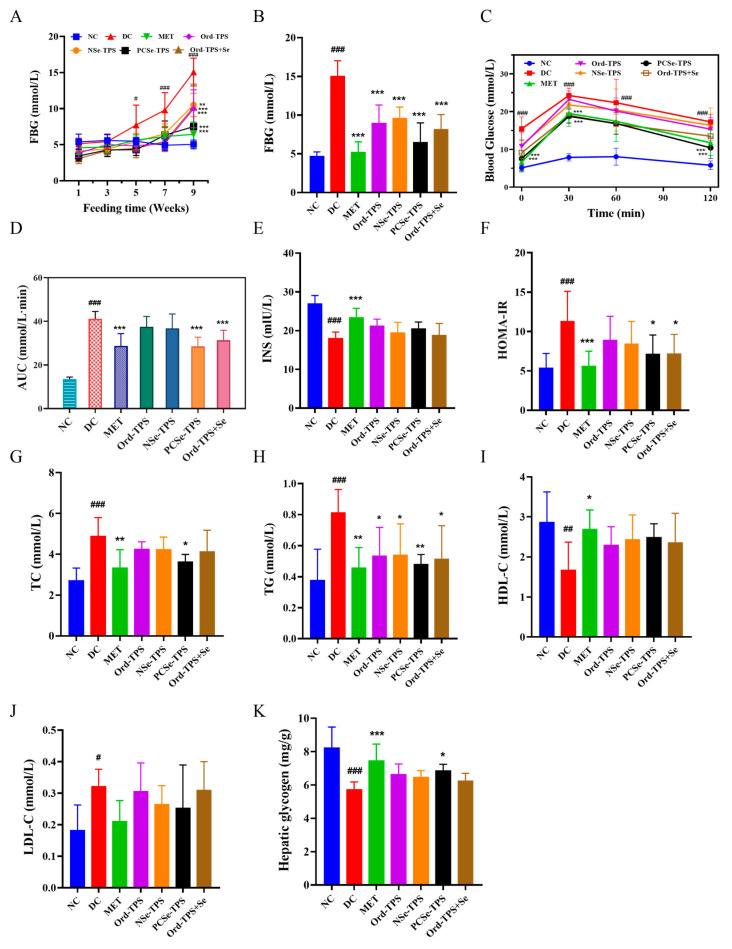
Effects of different selenium green tea polysaccharides on mice’s blood glucose and blood lipids (n = 8): (**A**) blood glucose changes, (**B**) FBG, (**C**) INS, (**D**) HOMA-IR, (**E**) oral glucose tolerance test (OGTT), (**F**) area under curve (AUC), (**G**) TC, (**H**) TG, (**I**) HDL-C, (**J**) LDL-C, and (**K**) hepatic glycogen. NC: normal control; DC: diabetes model control; MET: metformin positive control; Ord-TPS: ordinary tea polysaccharides; NSe-TPS: natural selenium-rich tea polysaccharides; PCSe-TPS: the chemically synthetic selenized tea polysaccharide with pulsed electric fields; Ord-TPS+Se: Ord-TPS and Na_2_SeO_3_ mixture. # *p* < 0.05, ## *p* < 0.01, ### *p* < 0.001, compared to normal control (NC) group, * *p* < 0.05, ** *p* < 0.01, *** *p* < 0.001 compared to diabetes model (DC) group.

The FBG levels of the model group were consistently higher than those of the other groups from the 5th week, indicating high-sucrose and high-fat diet-induced hyperglycemia (Figure 2A). By the 9th week, the FBG level of the DC group had reached 15.06 mmol/L, confirming the successful establishment of the diabetic model (Figure 2B). Different selenium tea polysaccharide interventions inhibited FBG elevation, and PCSe-TPS showed a better protective effect with lower FBG levels. In addition, the results of the OGTT demonstrated that the PCSe-TPS and Ord-TPS+Se interventions effectively improved glucose intolerance (Figure 2C). This was evidenced by the significantly lower AUC values compared to the control group (Figure 2D). Higher hepatic glycogen was also found in the PCSe-TPS groups than in the DC group (Figure 2K). This suggests that glucose in the blood is converted to synthesize liver glycogen. The HOMA-IR index was utilized to assess insulin resistance. The HOMA-IR level in the diabetic group was 110.00% higher than that in the NC group, indicating the occurrence of insulin resistance. Compared to the DC group, interventions with different selenium tea polysaccharides reduced the HOMA-IR levels, especially the PCSe-TPS and Ord-TPS+Se interventions, which showed significance (Figure 2F). It suggested that PCSe-TPS and Ord-TPS+Se could be beneficial for alleviating IR in the development of diabetic mice.

In addition, different selenium tea polysaccharides tended to counteract the increase in TG and LDL-C and the decrease in HDL-C; however, there was no statistical significance elicited by these groups (Figure 2G–J). Only PCSe-TPS significantly inhibited the TC content elevation in the development of diabetic mice (Figure 2G). It suggests that PCSe-TPS exhibits a significant effect in alleviating hyperlipidemia in the development of diabetic mice.

### 3.3. Different Selenium Tea Polysaccharides Enhanced Insulin Signaling Pathway PI3K/Akt

**Figure 3:**
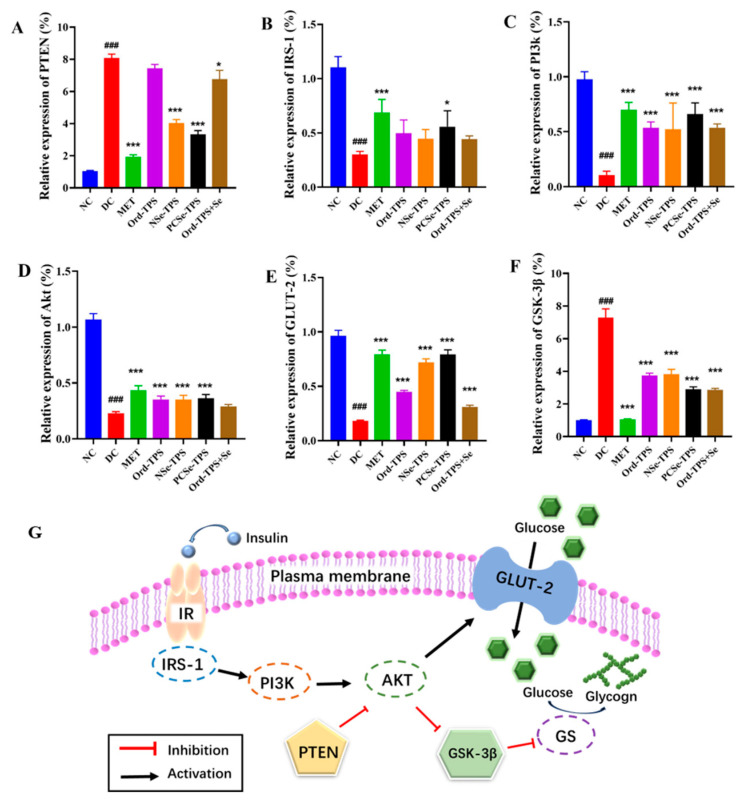
Effects of different selenium green tea polysaccharides on the expression of insulin resistance-related genes in mice (n = 4). (**A**) PTEN, (**B**) IRS-1, (**C**) PI3K, (**D**) Akt, (**E**) GLUT-2, (**F**) GSK-3β, (**G**) mechanisms of regulation. NC: normal control; DC: diabetes model control; MET: metformin positive control; Ord-TPS: ordinary tea polysaccharides; NSe-TPS: natural selenium-rich tea polysaccharides; PCSe-TPS: the chemically synthetic selenized tea polysaccharide with pulsed electric fields; Ord-TPS+Se: Ord-TPS and Na_2_SeO_3_ mixture. ### *p* < 0.001, compared to normal control (NC) group, * *p* < 0.05, *** *p* < 0.001 compared to diabetes model (DC) group.

The PI3K/AKT signaling pathway mediated by insulin is essential in the pathogenesis of diabetes mellitus [18]. To explore the possible mechanism of different selenium tea polysaccharides in alleviating blood glucose elevation and insulin resistance, we quantified the mRNA expression levels of key targets involved in insulin sensitivity, which included the PI3K/AKT pathway and its upstream components (phosphatase and tensin homolog (PTEN) and insulin receptor substrate 1 (IRS-1)) as well as downstream elements (glycogen synthase kinase-3β (GSK-3β) and glucose transporter-2 (GLUT-2)) (Figure 3G).

The mRNA expression levels of PTEN and GSK-3β were significantly increased, while IRS-1, PI3k, Akt, and GLUT-2 were decreased considerably in the DC group compared to the NC group (*p* < 0.001), demonstrating the dysregulation of the insulin-mediated PI3K/AKT pathway in diabetes. Selenium tea polysaccharide interventions, especially NSe-TPS and PCSe-TPS, prevented the down-regulation of these genes (Figure 3). Notably, only PCSe-TPS was found to significantly inhibit all of the above gene expression changes in the development of diabetic mice. These results suggested that PCSe-TPS could significantly improve blood glucose levels by regulating glucose uptake and metabolism through the PI3K/Akt pathway.

### 3.4. Different Selenium Tea Polysaccharides Reduced Liver Dysfunction, Oxidative Stress, and Inflammation

**Figure 4:**
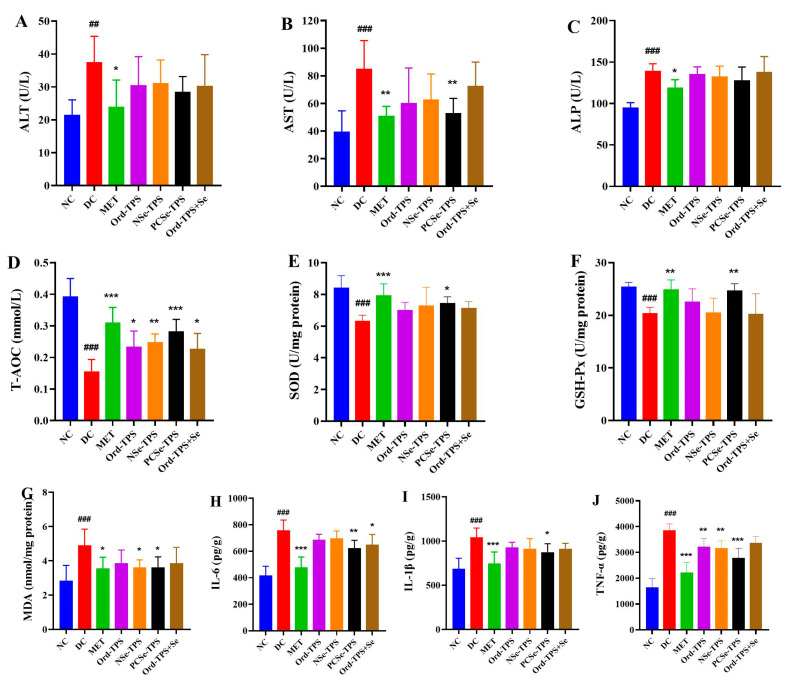
Effects of different selenium green tea polysaccharides on (**A**) ALT, (**B**) AST, (**C**) ALP, (**D**) T-AOC, (**E**) SOD, (**F**) GSH-PX, (**G**) MDA, (**H**) IL-6, (**I**) IL-1β and (**J**)TNF-α in the liver of mice (n = 8). NC: normal control; DC: diabetes model control; MET: metformin positive control; Ord-TPS: ordinary tea polysaccharides; NSe-TPS: natural selenium-rich tea polysaccharides; PCSe-TPS: the chemically synthetic selenized tea polysaccharide with pulsed electric fields; Ord-TPS+Se: Ord-TPS and Na_2_SeO_3_ mixture. ## *p* < 0.01, ### *p* < 0.001, compared to normal control (NC) group, * *p* < 0.05, ** *p* < 0.01, *** *p* < 0.001 compared to diabetes model (DC) group.

Persistent hyperglycemia and hyperlipidemia can cause liver dysfunction, oxidative stress, and inflammation [4]. Liver dysfunction, reflected by increased ALT, AST, and ALP activities, was observed in the DC group compared to the normal group (Figure 4A–C). However, treatment with MET and different selenium tea polysaccharides alleviated these elevated enzyme activities, with PCSe-TPS significantly inhibiting their increase. Oxidative stress was also observed in the DC group, indicated by decreased SOD, T-AOC, and GSH-Px activities and increased MDA levels. As expected, different selenium tea polysaccharides inhibited T-AOC decline and MDA elevation (Figure 4D,G). Notably, only PCSe-TPS showed a significant protective effect on alleviating the activities of SOD and GSH-Px (Figure 4E,F). Elevated pro-inflammatory factors in liver tissue are an essential factor leading to inflammation and liver dysfunction. Selenium tea polysaccharide interventions attenuated the high levels of IL-6, IL-1β, and TNF-α induced by a high-sucrose and high-fat diet combined with STZ. However, only PCSe-TPS showed significantly down-regulated effects on all these pro-inflammatory factors in the development of diabetic mice (Figure 4H–J). In summary, PCSe-TPS can potentially prevent the onset of diabetes by alleviating hepatic damage, oxidative stress, and inflammation.

### 3.5. Different Selenium Tea Polysaccharides Alter the Gut Microbiota in Diabetic Mice

Since T2D leads to dysbiosis of the gut microbiota and tea polysaccharides can regulate the structure of the gut microbiota [19], the effects of different selenium tea polysaccharides on the gut microbiota structure during the development of diabetic mice were examined.

**Figure 5:**
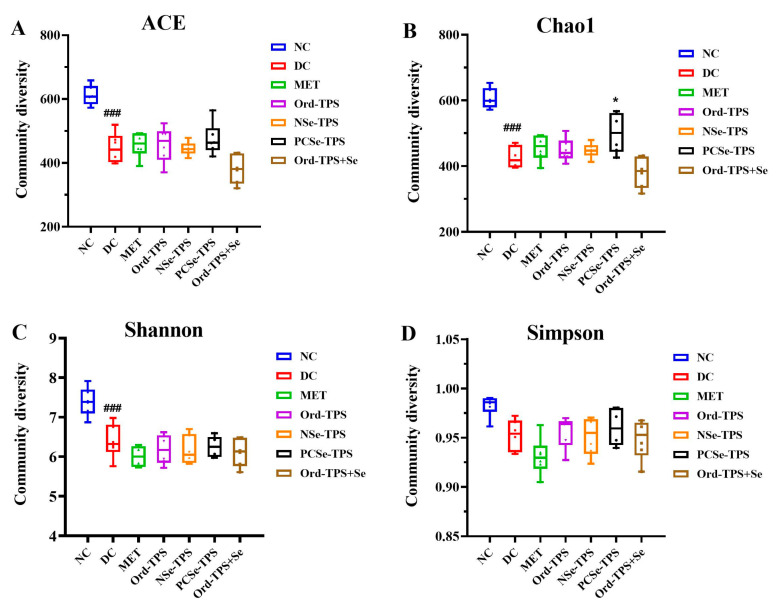
Effects of different selenium green tea polysaccharides on α-diversity of gut microbiota in mice (n = 6): (**A**) ACE, (**B**) Chao1, (**C**) Shannon, and (**D**) Simpson. NC: normal control; DC: diabetes model control; MET: metformin positive control; Ord-TPS: ordinary tea polysaccharides; NSe-TPS: natural selenium-rich tea polysaccharides; PCSe-TPS: the chemically synthetic selenized tea polysaccharide with pulsed electric fields; Ord-TPS+Se: Ord-TPS and Na_2_SeO_3_ mixture. ### *p* < 0.001, compared to normal control (NC) group, * *p* < 0.05 compared to diabetes model (DC) group.

The bacterial alpha diversity refers to species richness and evenness within a given ecosystem. As shown in Figure 5, the Ace, Chao1, Shannon, and Simpson indices decreased in the DC group compared to the NC group. Although no significant alterations were observed in the Ace, Chao1, Shannon, and Simpson indices between the intervention and DC groups, PCSe-TPS significantly prevented the reduction in the Chao1 index (Figure 5). This suggests that PCSe-TPS can potentially ameliorate the decline in bacterial species richness.

**Figure 6:**
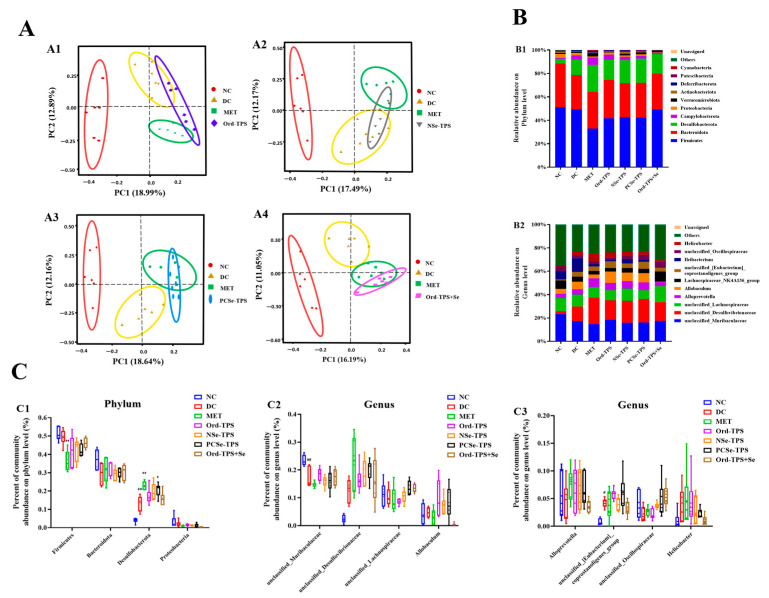
Effect of different selenium green tea polysaccharides on β-diversity and relative abundance of gut microbiota in mice (n = 6): (**A**) PCoA analysis based on binary Jaccard: (**A1**) NC, DC, MET and Ord-TPS groups; (**A2**) NC, DC, MET and NSe-TPS groups; (**A3**) NC, DC, MET and PCSe-TPS groups; (**A4**) NC, DC, MET and Ord-TPS+Se groups; (**B**) the gut bacterial composition at the (**B1**) phylum and (**B2**) genus level; (**C**) the relative abundance of some bacterial flora at (**C1**) phylum level and genus level of (**C2**) *unclassified_Muribaculaceae, unclassified_Desulfovibrionaceae, unclassified_Lachnospiraceae*, *and Allobaculum,* (**C3**) *Alloprevotella, unclassified_[Eubacterium]_coprostanoligenes_group, unclassified_Oscillospiraceae, and Helicobacter*. NC: normal control; DC: diabetes model control; MET: metformin positive control; Ord-TPS: ordinary tea polysaccharides; NSe-TPS: natural selenium-rich tea polysaccharides; PCSe-TPS: the chemically synthetic selenized tea polysaccharide with pulsed electric fields; Ord-TPS+Se: Ord-TPS and Na_2_SeO_3_ mixture. # *p* < 0.05, ## *p* < 0.01, compared to normal control (NC) group, * *p* < 0.05, ** *p* < 0.01, compared to diabetes model (DC) group.

PCoA based on OTUs revealed a distinct clustering of microbiota composition among the DC, MET, and NC groups (Figure 6A). This clustering indicated that the combination of a high-sucrose and high-fat diet with STZ injection had a significant impact on the structure of the gut microbiota community in mice, leading to gut microbiota dysbiosis. As anticipated, the dysbiosis of the gut microbiota was modulated by MET and different selenium tea polysaccharide interventions, although a slight overlap between the group ellipses of the DC and Ord-TPS, as well as the DC and NSe-TPS groups, was observed in the PCoA plot. The PCSe-TPS and Ord-TPS+Se intervention groups also displayed a similar clustering of the gut bacterial community relative to the MET group, indicating sound protective effects of PCSe-TPS and Ord-TPS+Se interventions.

The bacterial communities and their relative abundances were further investigated. At the phylum level, the DC group exhibited a significant increase in the relative abundance of *Desulfobacterota*, along with a decrease in the relative abundance of *Bacteroidota* and *Proteobacteria* compared to the NC group (Figure 6(B1)). Both the MET and selenium tea polysaccharide groups demonstrated a significant decrease in the relative abundance of *Firmicutes* and a considerable increase in the relative abundance of *Desulfobacterota* compared to the DC group. At the genus level, the relative abundances of *unclassified_Desulfovibrionaceae* and *Helicobacter* were higher, and the relative abundances of *unclassified_Muribaculaceae* and *unclassified_Oscillospiraceae* were lower in the DC group than those in the NC group (Figure 6(B2,C3)). The relative abundances of *unclassified_[Eubacterium]_coprostanoligenes_group*, *unclassified_Lachnospiraceae*, and *unclassified_Oscillospiraceae increased* in the Ord-TPS and PCSe-TPS intervention groups compared with those in the DC group. Furthermore, the relative abundance of *Allobaculum* increased in the Ord-TPS, NSe-TPS, and PCSe-TPS intervention groups but decreased in the Ord-TPS+Se intervention group compared with that of the DC group.

**Figure 7:**
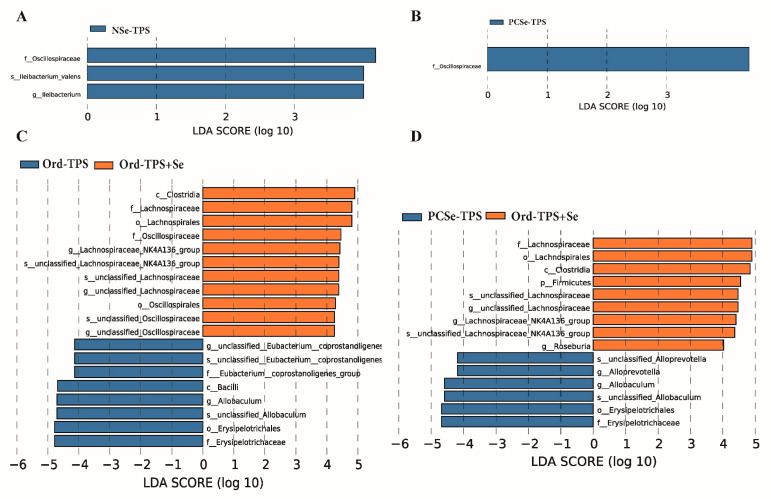
LEfSe analysis to identify taxonomic differences in the gut microbiota of the mice administrated with different selenium green tea polysaccharides (n = 6): (**A**) TPS and NSe-TPS, (**B**) TPS and PCSe-TPS, (**C**) TPS and Ord-TPS+Se, and (**D**) PCSe-TPS and Ord-TPS+Se (linear discriminant analysis score (log10) ≥ 4.0). NOrd-TPS: ordinary tea polysaccharides; NSe-TPS: natural selenium-rich tea polysaccharides; PCSe-TPS: the chemically synthetic selenized tea polysaccharide with pulsed electric fields; Ord-TPS+Se: Ord-TPS and Na_2_SeO_3_ mixture.

The LEfSe algorithm was applied to identify the specific microbial taxa that were enriched in mice with different selenium tea polysaccharides (Figure 7). Our results showed that, compared with Ord-TPS, NSe-TPS was associated with the enrichment of *Ileibacterium* (Figure 7A), PCSe-TPS was related to the enrichment of *Oscillospiraceae* (Figure 7B), and Ord-TPS+Se was associated with the enrichment of *Lachnospiraceae_NK4A136_group*, *unclassified-Lachnospiraceae*, and *unclassified_Oscillospiraceae* (Figure 7C). We also compared the specific microbial taxa that were enriched in mice with organic selenium tea polysaccharides (PCSe-TPS) and the mixture of inorganic selenium and tea polysaccharides (Ord-TPS+Se). This showed that PCSe-TPS was associated with the enrichment of *Erysipelotrichaceae*, *Allobaculum*, and *Alloprevotella*, while Ord-TPS+Se was related to the enrichment of *Firmicutes*, *Roseburia*, *unclassified-Lachnospiraceae*, and *Lachnospiraceae_NK4A136_group* (Figure 7D).

## 4. Discussion

In recent years, selenium polysaccharides have garnered interest due to their role as organic selenium sources and superior function to that of individual polysaccharides, such as hypoglycemic effects [11,12]. However, the protective effects of different selenium polysaccharides obtained through various selenization methods in the development of diabetes have not been comprehensively compared. Therefore, studies were carried out to compare the effects of ordinary selenium-enriched, synthetic selenized, and selenium-mixed polysaccharides from green tea on preventing the onset of diabetes.

The essential features of T2D include higher FBG levels, lower glucose tolerance and IR, and disturbances in glucolipid metabolism [4]. In our study, different selenium tea polysaccharides significantly prevented the upregulation of FBG. However, only PCSe-TPS and Ord-TPS+Se significantly prevented the upregulation of OGTT and HOMA-IR in the development of diabetic mice. This is related to the higher selenium content in PCSe-TPS and Ord-TPS+Se. In our work, the daily selenium intake of mice in the PCSe-TPS and Ord-TPS+Se groups was around 100 times higher than that of the NSe-TPS group. A previous study found that the selenized mycelial polysaccharide from *C. ventricosum* with a medium selenium content had the best anti-diabetic efficacy in mice [20]. Another study found that ordinary and selenium-enriched black tea showed similar glucose lowering effects in diabetic mice [10]. Insulin resistance can result in the dysfunction of the pancreas [21]. PCSe-TPS intervention significantly attenuated pancreatic lesions, and the intervention was best compared to the effects of the other polysaccharide groups. TC and TG levels in the blood reflect lipid metabolism [22]. Only PCSe-TPS significantly inhibited both TC and TG content elevation in the development of diabetic mice. The liver and kidney are vital metabolic organs, and the pancreas is the tissue that controls insulin secretion. As a form of inorganic selenium, Ord-TPS+Se showed its side effects by reducing body weight and increasing the organ index. Thus, the protective effects of PCSe-TPS were better in the essential characteristics of the development of diabetic mice compared to those of the other groups. It is related to the appropriate dose and form of selenium, as the toxicity tolerance dose and bioavailability of organic selenium are higher than that of inorganic selenium [8].

It is known that the abnormality of the PI3K-Akt signaling pathway is one of the main reasons for the onset of insulin resistance [4,9,23]. PTEN and IRS-1 are upstream molecules within the PI3K-Akt signaling pathway [24,25]. GLUT-2 and GSK-3β, which play crucial roles in glucose transport, absorption, and glycogen synthase activity, are regarded as downstream molecules within the PI3K-Akt signaling pathway [26]. In our study, the up-regulation of PTEN and GSK-3β and the down-regulation of IRS-1, PI3k, Akt, and GLUT-2 were effectively inhibited by different selenium tea polysaccharides, promoting liver glycogen synthesis and lowering blood glucose levels. Thus, different selenium tea polysaccharides protect against the development of diabetes in mice via the PI3K/Akt signaling pathway. However, only PCSe-TPS showed significance in regulating all the abovementioned genes. This suggests that the regulation effects of PCSe-TPS on the PI3K/Akt signaling pathway are better than those of the other selenium tea polysaccharides, which are consistent with the fundamental indices mentioned above (i.e., TC, hepatic glycogen, and pancreatic lesions (Figure 2 and Figure 3)). This was related to the different regulatory mechanisms of organic and inorganic selenium on the development of diabetic mice because of the different levels of remission exhibited.

Diabetes is associated with the disruption of antioxidant defense mechanisms due to low levels of antioxidant enzymes and excess free radicals under a hyperglycemic environment [27]. GSH-Px is an important peroxidative catabolic enzyme widely found in the organism. The active center of GSH-Px is selenocysteine, and the magnitude of its activity reflects the selenium level of the organism [28]. As expected, the administration of PCSe-TPS resulted in a significant increase in GSH-Px and SOD levels and a decrease in MDA levels. However, the effects of Ord-TPS+Se on these antioxidant enzymes’ levels were not statistically significant. It was also found that organic selenium supplementation exhibited higher erythrocyte GSH-Px activity than inorganic selenium supplementation [29]. Additionally, the activity of GSH-Px and the selenium content in the whole blood of lambs that were fed organic selenium (selenium-enriched yeast and selenium-enriched probiotics) were found to be higher compared to lambs that were fed sodium selenite [28]. This suggests that the antioxidant effect of organic selenium is more effective than inorganic selenium in vivo, which may be related to the bioavailability of selenium in different forms [8]. Our study revealed that the onset of oxidative stress is accompanied by inflammation, leading to increased levels of pro-inflammatory factors such as IL-6, IL-1β, and TNF-α in diabetic mice. Elevated levels of pro-inflammatory factors in liver tissue contribute to the inflammatory response that leads to liver dysfunction [30]. Serum ALT, AST, and ALP levels were considered as liver function damage markers [31]. As expected, PCSe-TPS intervention significantly decreased the levels of IL-6, IL-1β, and TNF-α, inhibited the elevation of ALT, AST, and ALP, and alleviated the liver tissue lesions in the development of diabetic mice.

Analysis of the fecal microbiota demonstrated that selenium tea polysaccharides significantly alleviated gut microbiota dysbiosis in the development of diabetic mice. Our findings demonstrated that although only PCSe-TPS exhibited an inhibitory effect on the decreased richness of gut microbiota in diabetic mice, all interventions of selenium tea polysaccharides caused alterations in the β-diversity of gut bacteria during the development of diabetes in mice. The abundance of *Oscillospira* was significantly reduced in the gut microbiota of diabetes mellitus patients [32]. In our study, the declining abundance of *unclassified_Oscillospiraceae* in the development of diabetic mice was reversed by Ord-TPS and PCSe-TPS intervention. *Unclassified_Oscillospiraceae* was found to possess the ability to synthesize short-chain fatty acids (SCFAs), specifically butyrate. SCFAs are essential in regulating energy metabolism and maintaining the intestinal environment’s homeostasis [33]. In addition, acetate and butyrate have been found to show a protective effect on β-cell survival in vitro [34]. Sodium-butyrate treatment indirectly increased insulin secretion, mainly by participating in glucagon-like peptide-1 (GLP-1) secretion [35]. GLP-1 promotes the secretion of insulin and has good hypoglycemic function. Thus, Ord-TPS and PCSe-TPS intervention could up-regulate the relative abundance of *unclassified Oscillospiraceae* to promote butyrate production and then enhance the secretion of GLP-1 to inhibit FBG elevation in the development of diabetic mice. The abundance of unclassified *Oscillospiraceae* and the level of GLP-1 were both found to be up-regulated in diabetic rats with pectic polysaccharide intervention [36]. Furthermore, compared with the DC group, the Ord-TPS and PCSe-TPS intervention up-regulated the relative abundance of *unclassified_[Eubacterium]_coprostanoligenes_group*, *unclassified_Lachnospiraceae*, and *Allobaculum*, while Ord-TPS+Se intervention decreased the relative abundance of *Allobaculum*. *Eubacterium_coprostanoligenes_group* and *Allobaculum* were identified as producers of SCFAs. It was observed that *Eubacterium_coprostanoligenes_group* demonstrated anti-dyslipidemia effects in response to a high-fat diet [37], while *Allobaculum* mitigated intestinal dysbiosis in diabetic mice [38]. *Unclassified Lachnospiraceae*, a potentially beneficial bacterium, was found to reduce the risk of T2D by reducing ketone body levels [39]. This indicates that different gut microbiota structures exist between different selenium tea polysaccharide interventions, although both alleviated gut microbiota dysbiosis in the development of diabetic mice. A previous study found that the relative abundance of *Ileibacterium* was significantly higher in the deacetylated konjac glucomannan group than in the konjac glucomannan group in mice fed a high-fat diet, although both exhibited hypolipidemic effects [40]. This suggests that modification of polysaccharides can change the gut microbiota structure regulation. Thus, we further identified the specific microbial taxa enriched in mice with different selenium green tea polysaccharides. Compared with the TPS group, the NSe-TPS group was characterized by *Ileibacterium*, the PCSe-TPS group was characterized by *Oscillospiraceae*, and the Ord-TPS+Se group was characterized by *Lachnospiraceae_NK4A136_group*, *unclassified-Lachnospiraceae*, and *unclassified_Oscillospiraceae*. These bacteria were found to be important in alleviating hyperglycemia and complications caused by diabetes [32,37,40]. Lastly, the gut microbiota structure in mice with organic and inorganic selenium was compared due to the different mitigating effects shown on the development of diabetes mellitus. *Erysipelotrichaceae*, *Allobaculum*, and *Alloprevotella* characterized PCSe-TPS, while Ord-TPS+Se was characterized by *Firmicutes*, *Roseburia*, *unclassified-Lachnospiraceae*, and *Lachnospiraceae_NK4A136_group*. *Erysipelotrichaceae* was found to be strongly associated with diabetic nephropathy [41]. *Erysipelotrichaceae UCG-003* increased in abundance in the healthy aging cohorts compared to the non-healthy aging cohorts [42]. *Alloprevotella*, *Allobaculum*, and *Roseburia* were reported to be SCFA producers [43,44]. Tea polysaccharides were found to restore the relative abundance of *Roseburia* and *Lachnospira* reduced by diabetes [19]. It suggests that synthetic selenylation alters the bioavailability of tea polysaccharides by the gut microbiota, which is related to the change in the structural properties of tea polysaccharides after selenylation [12,40].

## 5. Conclusions

Our study investigated the protective effects of different selenium green tea polysaccharides on the development of T2D in mice. The results showed that PCSe-TPS exhibited better protection against the development of T2D than NSe-TPS and Ord-TPS+Se. PCSe-TPS inhibited elevated blood glucose by regulating the PI3K/Akt signaling pathway and alleviated liver tissue oxidative damage and inflammatory responses. In addition, PCSe-TPS intervention reversed the decline in bacterial species richness and the abundance of *unclassified Oscillospiraceae* in the development of diabetes in mice. Our work provides a comprehensive understanding of the protective effects of different selenium tea polysaccharides on diabetes onset and lays the theoretical foundation for applying PCSe-TPS-related products.

## Figures and Tables

**Table 1:** Effects of different selenium green tea polysaccharides on organ index of mice.

Groups	Heart (%)	Liver (%)	Spleen (%)	Lungs (%)	Kidney (%)	Thymus (%)	Pancreas (%)	Stomach (%)
NC	0.52 ± 0.06	3.36 ± 0.29	0.21 ± 0.02	0.56 ± 0.03	1.03 ± 0.10	0.19 ± 0.05	0.61 ± 0.15	0.82 ± 0.13
DC	0.53 ± 0.09	4.99 ± 0.42 ^###^	0.24 ± 0.02	0.62 ± 0.05	1.31 ± 0.27 ^###^	0.18 ± 0.05	0.93 ± 0.05 ^###^	0.70 ± 0.19
MET	0.53 ± 0.05	4.25 ± 0.34 ***	0.26 ± 0.05	0.62 ± 0.04	1.12 ± 0.03 *	0.18 ± 0.06	0.72 ± 0.16 **	0.79 ± 0.18
Ord-TPS	0.56 ± 0.07	4.43 ± 0.51 *	0.27 ± 0.04	0.62 ± 0.04	1.15 ± 0.10 *	0.17 ± 0.03	0.82 ± 0.20	0.78 ± 0.09
NSe-TPS	0.55 ± 0.10	4.33 ± 0.31 **	0.23 ± 0.03	0.59 ± 0.03	1.12 ± 0.07 *	0.14 ± 0.03	0.81 ± 0.06	0.69 ± 0.09
PCSe-TPS	0.54 ± 0.07	4.13 ± 0.40 ***	0.26 ± 0.05	0.61 ± 0.06	1.11 ± 0.06 **	0.15 ± 0.04	0.79 ± 0.09	0.76 ± 0.06
Ord-TPS+Se	0.65 ± 0.11 ^##^	4.46 ± 0.57 *	0.49 ± 0.06 ^###^	0.77 ± 0.12 ^###^	1.17 ± 0.13	0.15 ± 0.06	0.82 ± 0.16	0.77 ± 0.10

Mean values ± standard deviation (n = 12). NC: normal control; DC: diabetes model control; MET: metformin positive control; Ord-TPS: ordinary tea polysaccharides; NSe-TPS: natural selenium-rich tea polysaccharides; PCSe-TPS: the chemically synthetic selenized tea polysaccharide with pulsed electric fields; Ord-TPS+Se: Ord-TPS and Na_2_SeO_3_ mixture. ## *p* < 0.01, ### *p* < 0.001, compared to normal control (NC) group, * *p* < 0.05, ** *p* < 0.01, *** *p* < 0.001 compared to diabetes model (DC) group.

## Data Availability

The data used to support the findings of this study can be made available by the corresponding author upon request.

## Supplementary Materials

The following supporting information can be downloaded at: https://www.mdpi.com/article/10.3390/foods12234190/s1, Figure S1: Experimental design; Figure S2: Protective effects of different doses of synthetic selenized green tea polysaccharides (PCSe-TPS) on the development of diabetic mice; Table S1: Reaction system of RT-qPCR; Table S2: Reaction procedure of RT-qPCR; Table S3: Primer sequences.
