# Supplementary material for: Protective Effects of Different Selenium Green Tea Polysaccharides on the Development of Type 2 Diabetes in Mice
Source: Foods. 2023 Nov 21;12(23):4190. doi: 10.3390/foods12234190 (PMC10706338; doi:10.3390/foods12234190)
Supplement: Supplementary file 1 [file foods-12-04190-s001.zip › foods-2692855-supplementary.pdf]

Table S1 Reaction system of RT-qPCR

Composition	Volume (μL)
F-primer (10 μM)	0.4
R-primer (10 μM)	0.4
2×Taq Pro Universal SYBR qPCR Master Mix	10
Template DNA/cDNA	2
Nuclease-free water	To 20

Table S2 Reaction procedure of RT-qPCR

Steps	Temperature (℃)	Time (s)	Number of cycles
Pre-denaturation	95	30	1
Denaturation	95	10	40
Annealing/extension	60	30	
Melting curve collection	95	15	1
	60	60	
	95	15	

Table S3 Primer sequences

Gene	Primer	Sequence (5'-3')	Length
β-actin	Forward	CACGATGGAGGGGCCGGACTCATC	240 bp
	Reverse	TAAAGACCTCTATGCCAACACAGT	
PTEN	Forward	GAAAGGGACGGACTGGTGTA	199 bp
	Reverse	AGTGCCACGGGTCTGTAATC	
IRS-1	Forward	ATTAACCCCATCAGACGCCA	210 bp
	Reverse	AGGAGGTTTGGCATGAGGAA	
PI3K	Forward	AATGCACGGCGATTACACTC	199 bp
	Reverse	GGACACTGGGTAGAGCAACT	
Akt	Forward	CTGCCCTTCTACAACCAGGA	214 bp
	Reverse	CATACACATCCTGCCACACG	
GSK-3β	Forward	AGCCACTGATTACACGTCCA	243 bp
	Reverse	AAATGTCCTGCTCCTGGTGA	
GLUT-2	Forward	AGTTCGGCTATGACATCGGT	234 bp
	Reverse	ACACAGACAGAGACCAGAGC	

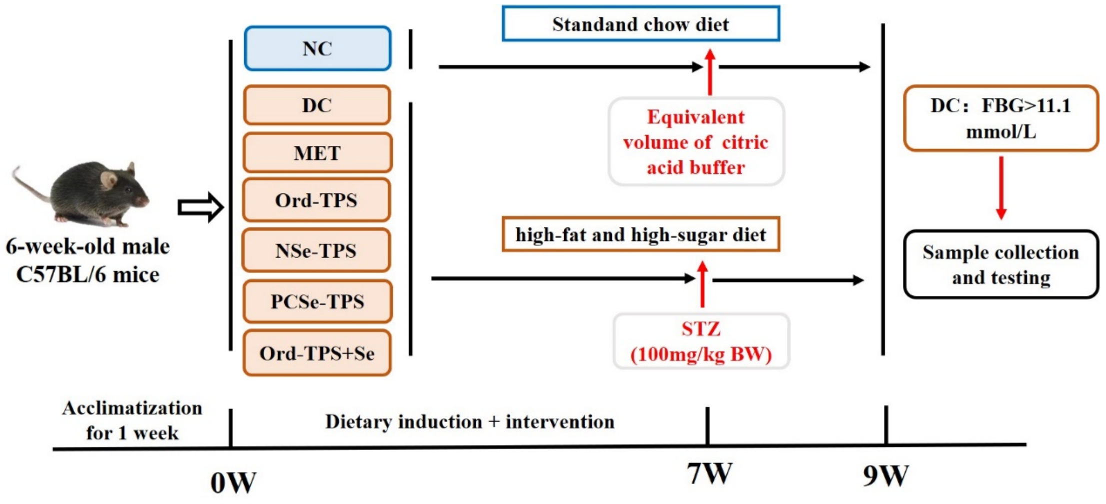

Figure S1. Experimental design. After acclimatization for one week, 7-week-old male C57BL/6J mice were divided into seven groups (n=12): control (NC), model (DC), positive control (MET), Ord-TPS, NSe-TPS, PCSe-TPS, and Ord-TPS+Se. The normal control (NC) group was fed a basic diet, and the other groups were fed a high-sucrose and high-fat diet. The positive control group (MET) was gavaged with metformin, and the intervention groups were gavaged with different selenium tea polysaccharides (200 mg/kg) for six weeks. At the 7th week, the mice were injected with streptozotocin (STZ, 100 mg/kg body weight) for two consecutive days. In the 9th week, fasting blood glucose (FBG) was measured, and mice with FBG levels > 11.1 mmol/L were considered diabetic mice. Then, the effects of different selenium green tea polysaccharides on the diabetes-related biomarkers and gut microbiota were evaluated.

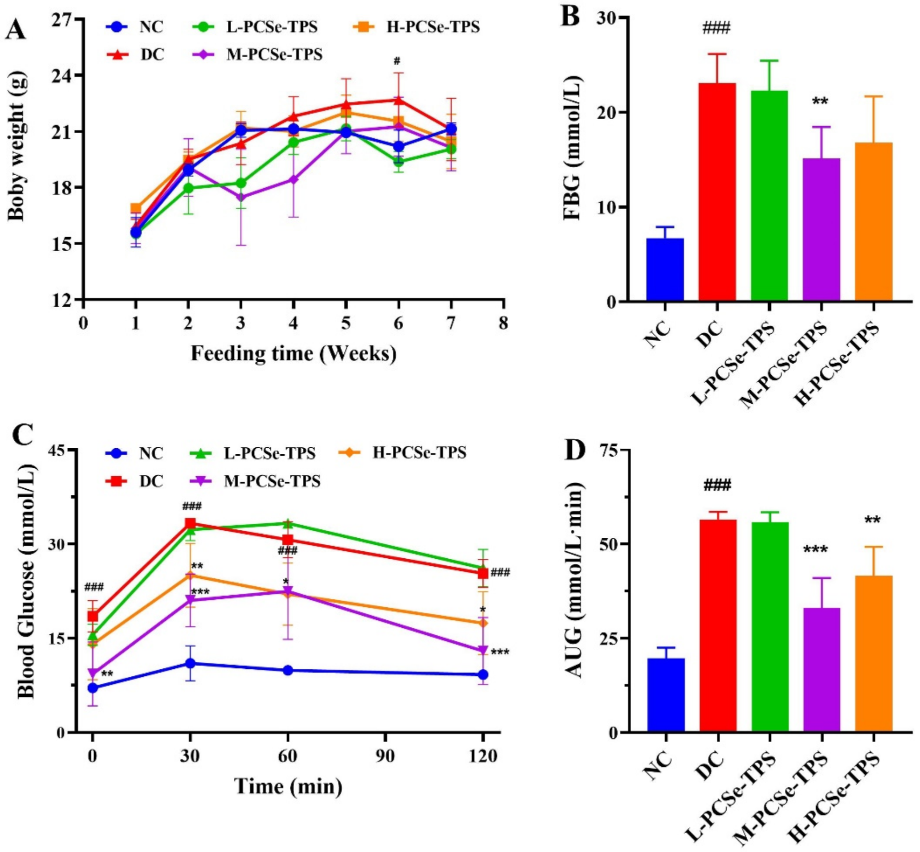

Figure S2 Protective effects of different doses of synthetic selenized green tea polysaccharides (PCSe-TPS) on the development of diabetic mice. (**A**) Changes in body weight, (**B**) FBG, (**C**) OGTT, (**D**) Area under the curve. NC: normal control group; DC: diabetes model control group. L-PCSe-TPS: low-dose PCSe-TPS group, 100 mg/kg; M-PCSe-TPS: medium-dose PCSe-TPS group, 200 mg/kg; H-PCSe-TPS: high-dose PCSe-TPS group, 400 mg/kg. # p<0.05, ### p<0.001, DC versus NC; ** p<0.01, *** p<0.001, compared to the diabetes model (DC) group.
